# Myasthenia gravis and myopathy after nivolumab treatment for non‐small cell lung carcinoma: A case report

**DOI:** 10.1111/1759-7714.13177

**Published:** 2019-08-21

**Authors:** Je‐Seong Kim, Tai‐Seung Nam, Jieun Kim, Bo‐Gun Kho, Cheol‐Kyu Park, In‐Jae Oh, Young‐Chul Kim

**Affiliations:** ^1^ Department of Internal Medicine Chonnam National University Medical School, and Chonnam National University Hwasun Hospital Hwasun South Korea; ^2^ Department of Neurology Chonnam National University Medical School and Chonnam National University Hospital Gwangju South Korea

**Keywords:** Myasthenia gravis, myopathy, nivolumab, NSCLC

## Abstract

Here, we report a case of myasthenia gravis and myopathy in a patient treated with nivolumab. A 76‐year‐old man who had been treated with four doses of nivolumab because of non‐small cell lung cancer (NSCLC) presented with proximal‐dominant muscle weakness and fluctuating ptosis and diplopia. Serologic studies revealed increased levels of muscle enzymes including creatine phosphokinase (2934 U/L), and acetylcholine receptor antibody was positive (1.31 nmol/L). Following electrodiagnostic study, he was diagnosed with myasthenia gravis and active stage of myopathy. After discontinuation of nivolumab, he was treated with corticosteroids, intravenous immunoglobulin G, and pyridostigmine. The neuromuscular symptoms and serologic abnormalities of the patient markedly improved. Currently, he is taking oral steroids and pyridostigmine without further immunotherapy.

## Introduction

Immune checkpoint inhibitors (ICIs) inhibit the programmed cell death (PD)‐1/PD‐ligand 1 (PD‐L1) pathway and thus block T cells from being inactivated. Nivolumab, pembrolizumab, and ipilimumab are broadly used in cancers such as melanoma, non‐small cell lung cancer (NSCLC), and urothelial carcinoma.^1^, ^2^, ^3^ They rarely cause fatal side effects, but recently, specific immune‐related adverse events (irAEs) involving neuromuscular systems have been reported.^4^ Several cases of myasthenia gravis with myopathy have already been published, but the exact mechanisms of these conditions are still unclear.^5^, ^6^, ^7^, ^8^, ^9^, ^10^, ^11^, ^12^, ^13^ Here, we report a case of myasthenia gravis and acute myopathy in a patient treated with nivolumab.

## Case report

**Figure 1:**
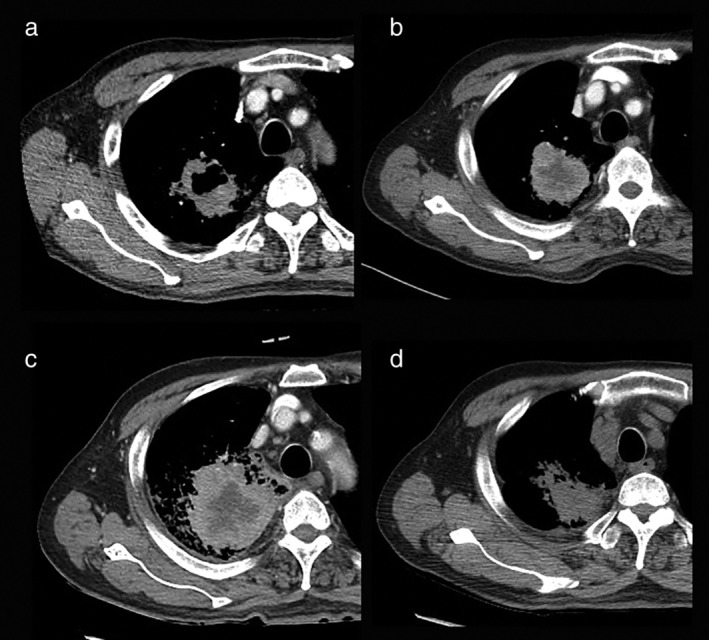
Representative chest computed tomography images (**a**) at diagnosis, (**b**) before administration of nivolumab, (**c**) after the first dose and (**d**) after the third dose of nivolumab treatment when the size of the lung mass and the ground‐glass opacities around the mass had decreased dramatically.

A 76‐year‐old man visited our hospital with a 4.8 cm diameter cavitary mass in the right upper lung field diagnosed on chest computed tomography (CT) (Fig 1a). A consolidation, which invaded the right pleura, was also observed, and transthoracic needle biopsy revealed non‐small cell lung cancer (NSCLC). Positron emission tomography CT showed right pleural involvement, which suggested right pleural seeding, and the TNM staging was recorded as stage IVA (cT4N0M1a). He had stopped cigarette smoking 30 years ago. Epidermal growth factor receptor mutation and anaplastic lymphoma kinase rearrangements were negative, but PD‐L1 immunohistochemistry (SP‐263) was strongly positive with 75% tumor proportion score.

From April 2018, he received seven cycles of gemcitabine combined with carboplatin, but disease progression was noted after the seventh cycle (Fig 1b). After the first cycle of nivolumab (3 mg/kg), in September 2018, he had fever and chills with increase in size of the right lung mass, which was considered a sign of pseudoprogression (Fig 1c). After the third dose, the size of the lung mass and the ground‐glass opacities around the mass decreased dramatically (Fig 1d). The last and fourth dose was administered on 16 October 2018, but the patient subsequently visited the emergency center on 19 October 2018 complaining of gait disturbance.

A neurologic examination revealed bilateral symmetric muscle weakness (Medical Research Council grade, III) of both legs and positive Gowers' sign. Additionally, ptosis and binocular diplopia were noted in the right eye, and bulbar symptoms including dysarthria and dysphagia were also observed. Magnetic resonance imaging of the brain was unremarkable. Serologic studies revealed significantly increased levels of muscle enzymes including creatine kinase (CK; 2934 U/L, normal level, NR < 187), aspartate aminotransferase (231 U/L, NR < 38), alanine transaminase (231 U/L, NR < 42), myoglobin (3009 ng/mL, NR < 92.5), and lactate dehydrogenase (LDH, 1807 IU/L, NR < 472).

The levels of troponin I and CK myocardial isoform were also increased (0.408 ng/mL, NR < 0.05, and 73.8 ng/mL, NR <3.6, respectively), but electrocardiography revealed no significant changes when compared to the previous recordings. Acetylcholine receptor (AChR) antibody was positive (1.31 nmol/L, NR < 0.5), but thyroid function was normal, and the rest of the immunological work‐up including antinuclear antibodies, antineutrophil cytoplasmic antibodies, and anti‐Jo‐1 antibody were all negative.

**Figure 2:**
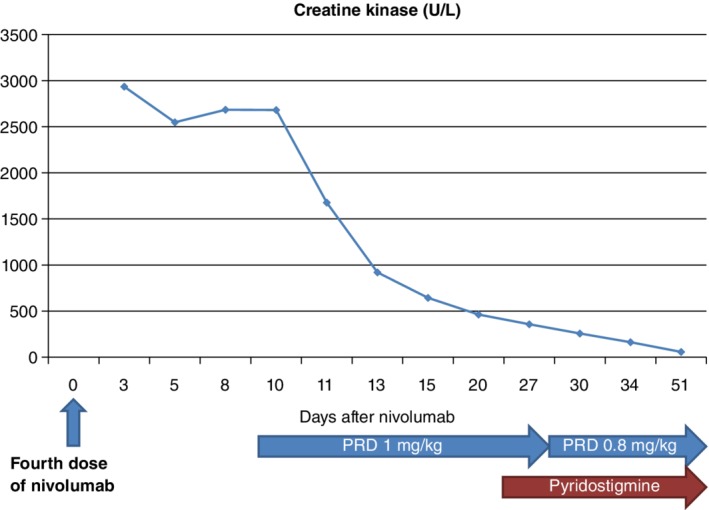
Following administration of the fourth and final dose of nivolumab, the patient's creatine kinase (CK) level was 2934 U/L (normal level, NR < 187). Intravenous corticosteroids (methylprednisolone 1 mg/kg/day) were administered and the CK level returned to its normal range by the 32nd day. Pyridostigmine was administered to relieve the patient's remaining symptoms at a dose of 30 mg three times a day for six days and this was increased to 60 mg three times a day. Intravenous steroids were tapered to oral prednisolone at 40 mg twice a day. PRD, prednisolone.

Hydration with normal saline was initiated, but his CK level remained high at 2765 U/L after a week. Intravenous corticosteroids (methylprednisolone 1 mg/kg/day) were administered, and the patient's symptoms improved and muscle enzyme levels decreased. CK level was in its normal range on the 32nd day (Fig 2), and the levels of myoglobin and LDH also significantly decreased to 259 ng/mL and 787 IU/L, respectively. However, the patient could still not walk without assistance.

Nerve conduction study and repetitive nerve stimulation test were unremarkable, but needle electromyography revealed prominent myopathic change with active denervation potentials including positive sharp waves and fibrillation. Pyridostigmine was administered to relieve the remaining symptoms at a dose of 30 mg three times a day for six days and was increased to 60 mg three times a day. The intravenous steroids were tapered to oral prednisolone at 40 mg twice a day. Gait disturbance improved steadily, and he could walk with the assistance of the walker.

The patient's difficulty in swallowing, dyspnea, and weakness of the legs had persisted for two months. Pulmonary function test revealed significantly decreased forced vital capacity (FVC, 1.71 L, 41.6%) compared to the previous test (FVC 3.58 L, 87%) performed before the onset of neurologic symptoms. Intravenous immunoglobulin was administered (400 mg/kg bodyweight) for five consecutive days, and the symptoms were gradually relieved. After one month, AChR‐binding antibodies decreased to 1.19 nmol/L, from the previous level (1.31 nmol/L).

While the patient remained on maintenance steroid treatment, 6000 cGy of stereotactic body radiation treatment to the primary tumor in the right upper lobe was administered in February 2019. He has not received nivolumab for more than eight months, but the primary tumor and metastatic lesions were still in their remission state. His motor symptoms improved, but general muscle weakness remained. His FVC was measured in April 2019 and had improved to 2.64 L (65.7%). He is on outpatient follow‐up while receiving prednisolone and pyridostigmine.

## Discussion

Targeting lymphocyte receptors or their ligands to enhance endogenous antitumor activity is a recent strategic approach in cancer control. PD‐1 inhibitors, a new class of drugs that block PD‐1, activate the immune system to attack tumors and are used to treat certain types of cancer.^14^

However, ICIs unbalance the immune system and generate dysimmune toxicities, known as irAEs. The incidence of irAEs with any grade is reported to range from 15% to 90%, and the rate of severe irAEs requiring discontinuation of the immunotherapy is estimated to range from 0.5% to 13%. The skin and gastrointestinal tract were mostly affected in 44% (95% confidence interval [CI], 38–49.5) and 35% (95% CI, 29–41) of cases, respectively, while endocrine and hepatic organs were less affected in 6% (95% CI, 4–8) and 5% (95% CI, 2–7) of cases, respectively. Other events such as neurologic diseases were rarely reported.^4^

Myasthenia gravis is an autoimmune disease caused by antibodies that bind to acetylcholine receptors, or to molecules in the postsynaptic membrane at the neuromuscular junction.^15^ The diagnosis of myasthenia gravis is established by the presence of specific autoantibodies and related symptoms.^16^ Ocular symptoms are the most common symptoms, and greater than 50% of patients present with ptosis or diplopia,^17^, ^18^ but the neck and limb muscles can also be involved.^19^, ^20^ Respiratory muscle weakness is considered the most fatal symptom among the reported symptoms of myasthenia gravis.^21^

In this case report, acute myopathy and myasthenia gravis was noted. The combination of these two conditions suggests very rare dysimmune toxicity, and several cases of myasthenia gravis with hyperCKemia have been reported worldwide.^5^, ^6^, ^7^, ^8^, ^9^, ^10^, ^11^, ^12^, ^13^ It is postulated that the simultaneous formation of AChR antibody and anti‐striational antibodies is required to develop myasthenia gravis with myositis. Anti‐striational antibodies react with epitopes on the muscle protein titin, abundant in the skeletal and cardiac sarcomere. It is known that anti‐titin antibody is accompanied with myositis.^22^ Moreover, myasthenia gravis with hyperCKemia is suspected to have a more complicated prognosis than in myasthenia gravis without hyperCKemia; thus, before ICI treatment, AChR antibodies and anti‐striational antibodies should be measured if available.^23^, ^24^ There is a possibility that patients already have subclinical myasthenia gravis, and ICIs are considered the triggering factors of flare‐up, but more studies are needed to confirm this hypothesis.

Several neurologic prognoses have been reported after the discontinuation of ICIs.^25^, ^26^, ^27^ It is difficult to reconsider causative immunotherapy if myasthenia gravis improves, as there are insufficient data about the safety of reintroducing ICIs after recovery. Meanwhile, more than half of the patients who experienced neuromuscular adverse events had combined other organs' adverse effects, such as thyroiditis, hepatitis, and colitis.^28^ A series of case reports about irAEs led to the introduction of clinical practice guidelines.^29^ Corticosteroid administration and discontinuation of ICIs are the core treatments, and alternative options also include intravenous immunoglobulin, cyclosporine A, cyclophosphamide, infliximab, mycophenolate mofetil, and plasmapheresis. Recently, cases of immune‐related myocarditis that were treated with alemtuzumab or abatacept have also been reported.^30^, ^31^

Electrodiagnostic study including nerve conduction study and electromyogram and serologic tests including the measurement of AChR antibodies and the levels of muscle enzymes should be performed promptly when patients complain of muscle weakness after receiving ICIs. Clinicians need to pay careful attention to subtle changes because symptoms that indicate neurologic complications such as myasthenia gravis can lead to death if diagnosed too late.
